# Letter from Dr. Blaney

**Published:** 1845-06

**Authors:** J. V. Z. Blaney

**Affiliations:** St. Louis


*St. Louis, May* 10, 1845.

D. Brainard, M.D.,

*Dear Sir*,—I arrived here yesterday morning, having been detained some time at Peoria, waiting the departure of a steamboat. Upon the steamboat in which I left Peoria for this city, I was pleased to find, as a fellow passenger, Dr. Henry, of Bloomington, Ills., whose letter on Epidemic Erysipelas, you will remember, was copied into a number of our last vol. I found Dr. H., as I had anticipated from his reputation, a gentlemen in manners and a scholar in acquirement. At the various points of stoppage on the Illinois river, whenever the detention of our boat for the receipt or delivery of freight or passengers permitted, Dr. H. and I embraced the opportunity of extending our acquaintance among the medical practitioners. I was much pleased to observe the high tone of the profession, and the number of medical gentlemen, evidently of excellent early education; and, from their still continuing, as far as the duties of their practice will permit, to be *reading* men, of much intelligence and acquirement.

The town of Pekin was of particular interest, as the point at which the Epidemic Erysipelas raged with most violence, some time since. From the account of the physicians of the place, it would appear, that one in every nine persons of the whole population was attacked, and that a large majority of the cases were fatal. Puerperal women and young children were peculiarly subject to attack, and among them principally the mortality existed. At this place I had the pleasure of making the acquaintance of Dr. Henry, who has lately removed from Springfield. The Dr. has promised me a paper on the Treatment of "Congestive Fever." The principal point of novelty in the mode of treatment which he professes to have originated, is the use of unusually large doses of opium.

The Epidemic Erysipelas has not yet entirely disappeared on the Illinois. Dr. Bond of Meredosia, Morgan Co., informed me that he had cases under treatment at the time of our interview.

*Medical Schools and Journals in St. Louis.*—I called yesterday upon several of the Professors in the Medical Schools. The classes in the two rival institutions—the St. Louis University and the Kemper College—were about equal in number. This was a gain on the part of the University over its classes of previous years, and rather a decline in the usual class of Kemper College. The riot which occurred last year, and which resulted in the destruction of part of the museum of the University, does not seem to have affected that institution more than its rival. The decline in the class of Kemper College is attributed, by its faculty, to the unfortunate introduction of Erysipelas into the class of '43 and '44, by contagion, in the anatomical rooms, from subjects which had died of that disease. I had but the time and opportunity to view the buildings externally. The new building of the University presents really a beautiful front, and from a description which has been given me of the internal arrangements, I should judge that it was remarkably convenient for professors and pupils, and admirably adapted to the purpose for which it was erected.

There are now published in St. Louis two Medical Journals. One, which I had not before seen or heard of, I saw upon the table of a medical gentleman. It has recently been issued, and is the organ of the Kemper College. As I did not obtain a number, and had not the honor of making the acquaintance of its editor, I can say but little of it. It will probably be received by you in exchange before this letter arrives. The other, the St. Louis Medical and Surgical Journal, has reached its third volume. It is much enlarged and improved. Dr. Wm. McPheters is associated as editor with Dr. Linton, the former editor. The combined industry and talent of these two gentlemen, will doubtless, make it all that a medical journal should be. It professes not to support the exclusive interests of either medical school, but to be devoted solely to the claims of the profession at large.

As I leave to-morrow, I will not have time to visit the Hospital. I will write again from Cincinnati, if my stay in that city will permit. Until then, I remain

Yours truly,

J. V. Z. BLANEY.

